# Contrasting niche dynamics in the invasion processes of two congeneric dinoflagellates

**DOI:** 10.1038/s41598-025-13849-9

**Published:** 2025-08-10

**Authors:** Rafael Lacerda Macêdo

**Affiliations:** 1 Leibniz Institute of Freshwater Ecology and Inland Fisheries (IGB), Müggelseedamm 310, 12587 Berlin, Germany; 2 Institute of Biology, Freie Universität Berlin, Königin-Luise-Str. 1-3, 14195 Berlin, Germany

**Keywords:** Biological invasions, Dinophyceae, Invasion mechanisms, Niche dynamics, Niche partitioning, Biogeography, Climate-change ecology, Conservation biology, Ecological modelling, Evolutionary ecology, Freshwater ecology

## Abstract

Niche-based models are essential for predicting invasion risks. Although most invasive species tend to conserve their ecological niches after introduction, some challenge this assumption by expanding or contracting their niches, yet such patterns remain underexplored in microorganisms. Since larger niche shifts can reduce the predictive performance of these models, this study examines whether the climatic niches of the invasive dinoflagellates *Ceratium hirundinella* (Müller, 1841) and *C. furcoides* (Langhans, 1925) have shifted following their invasion from native European to non-native American ranges, where they have caused significant impacts on biodiversity and water quality. Though both species are native to temperate European lakes, their colonization patterns in the Americas differ, and the drivers of their spread remain unclear. In this study, niche conservatism was analyzed using five niche dynamic metrics for both species. The current distribution of *C. hirundinella* primarily in subtropical and temperate non-native areas aligns with its native climate (i.e., higher niche stability), suggesting preadaptation. Meanwhile, for *C. furcoides*, a niche shift—indicated by maximum expansion and unfilling—suggests a much higher potential for rapid spread across both tropical and subtropical climates. These findings show distinct climatic responses of congeneric species in non-native ranges, emphasizing the need to move beyond native environmental predictors when assessing invasion risk. Future research should explore niche shifts over time and whether invasions begin in ecologically matched habitats (as expected for *C. hirundinella*) or are driven by propagule pressure and human activity despite niche mismatches (as in *C. furcoides*).

**Supplementary Information:**

The online version contains supplementary material available at 10.1038/s41598-025-13849-9.

## Introduction

The niche conservatism hypothesis suggests that species tend to retain their ecological niches across space and time^1^, forming a conceptual basis for predicting species distributions, understanding biogeographical patterns, and reconstructing evolutionary histories^1,2^. However, biological invasions represent an opportunity to revisit this hypothesis^3,4^. Invasive species may either conserve or shift their niches depending on ecological and evolutionary processes, including adaptive evolution, residence time, altered biotic interactions, or dispersal constraints^5,6^. Some studies report that niche conservatism is commom among invasive species^7,8^, although the available evidence is limited by overlooked taxonomic groups. Critically, the reliability of transferring species distribution models (SDMs) across regions or timeframes—niche transferability—depends on the extent of niche stability, and must be carefully evaluated rather than assumed, especially when projecting invasion risk^4,9^. This study contributes to this discussion by comparing *Ceratium furcoides* (Levander) Langhans 1925 and *C. hirundinella* (O.F. Müller) Dujardin 1841, two congeneric dinoflagellates with contrasting distribution patterns in their non-native ranges, to assess whether their spread reflects niche conservatism or change. It also critically evaluates the performance and assumptions of existing potential distribution models for these taxa.

*Ceratium furcoides* and *C. hirundinella* are bloom-forming invasive species that have rapidly spread across freshwater bodies, primarily in the Americas but also in regions like South Africa and China. These species are notorious for causing significant ecological and economic impacts in the areas where they have been introduced^10–12^. These freshwater dinoflagellates were first described in temperate European lakes^13,14^, which are assumed to be their native range where they share some morphological and ecological characteristics^15^. Strikingly underrepresented in the invasion science literature, other *Ceratium* species are known to occur in inland waters, although they are not as relevant as *C. furcoides* and *C. hirundinella* when it comes to biological invasions. This is partly due to biogeographical bias (e.g., microorganisms are thought to be ubiquitous)^16^ or to misidentification, which is not rare given the taxonomic complexity of microorganisms^17,18^. Although not yet fully understood, the invasion processes of *C. furcoides* and *C. hirundinella* occurred at different times and followed distinct geographic trajectories across the American continent, where the majority of reports have been made. *C. furcoides* has been documented as a later “newcomer,” colonizing areas previously unoccupied by either species, with species replacement and low co-occurrence being observed^19–21^. Studies have also emphasized the role of cyst banks, which enable blooms of active forms after nutrient concentrations increase following macrophyte removal. Although mono-specific blooms have been reported during both wet and dry seasons^21,22^, it is unclear whether these patterns reflect shared or divergent ecological requirements in their non-native ranges, and the environmental constraints shaping the distribution of these two species remain unclear^23,24^. Addressing these gaps is essential to determine how well native-range information predicts their invasive potential.

Recent evidence challenges the assumption that *C. furcoides* maintains its climatic niche between its native and invaded ranges, indicating spatial niche shift and future range expansion under climate change^25,26^. However, a more comprehensive understanding is required, particularly through the use of complementary statistical methods, such as the decomposition of niche changes into distinct components: niche stability (the proportion of the invaded niche that overlaps with the native niche), niche unfilling (the portion of the native niche not overlapping with the invaded niche), and niche expansion (the portion of the invaded niche that does not overlap with the native niche)^7^. Additionally, examining large-scale distribution can provide observation-based climate tolerance information on invasive species, enabling the testing of hypotheses on similar invasion patterns among closely related taxa (i.e., congeneric, convergent, sympatric species)^27,28^. Although congeneric species typically share similar environmental requirements due to their close phylogenetic relationship^29^, the distinct colonization patterns in non-native areas shown by *C. furcoides* and *C. hirundinella* support the hypothesis that significant differences in their niche dynamics may exist. Furthermore, a plausibly reduced niche overlap may minimize direct competition between these congeneric species, facilitating independent adaptation to local environmental conditions^30–32^. This process aligns with findings that environmental niche separation can promote coexistence by reducing interspecific competition^31^ and that niche partitioning may reflect trade-offs between adaptation to local environments and competitive interactions^32^.

Using *C. furcoides* and *C. hirundinella* as model organisms, this case study tests the hypothesis of niche conservatism to explore the role of niche dynamics in the spread of potentially harmful phytoplanktonic species. Specifically, it quantifies the degree of niche changes (e.g., stability, unfilling, and expansion), building upon previous findings of low niche overlap between native and invasive populations of *C. furcoides*^25^. In addition to shedding light on invasion mechanisms among closely related taxa, these analyses can inform predictive models and management strategies for invasive species that are virtually impossible to eradicate. They also provide a basis for future hypothesis-driven studies—such as whether reduced niche overlap between native and introduced populations contributes to broader, more persistent colonization and long-term co-occurrence through niche differentiation.

## Materials and methods

### Species data and environmental variables

Data on native and invaded areas were gathered from the Global Biodiversity Information Facility for *C. furcoides* (GBIF; 10.15468/dl.2zpm2w) and *C. hirundinella* (GBIF; 10.15468/dl.s3ey9w). The occurrence records were later thinned with a 10 km radius using the spthin library^33^. Processed data resulted in 273 records of *C. hirundinella* and 158 of *C. furcoides* from both native and non-native ranges (Table S1). Environmental predictors were obtained from the Worldclim database (https://www.worldclim.org/;^34^ with a 10 arcmin spatial resolution. This dataset includes a total of 19 bioclimatic variables derived from the monthly temperature and rainfall values such as mean annual temperature, annual precipitation and limiting environmental conditions (e.g., temperature of the coldest and warmest month). These variables are considered appropriate proxies for aquatic environments, supported by evidence that air temperature explains much of the variance in water temperature^35^, which is relevant for phytoplankton proliferation^36,37^, and that precipitation is an important driver of phytoplankton-limiting nutrient enrichment in freshwater catchments^38^. Thus, climate data have been proven useful in predicting the large-scale potential distributions of non-native phytoplankton species with high reliability^39–41^.

### Multivariate niche analysis

**Fig. 1:**
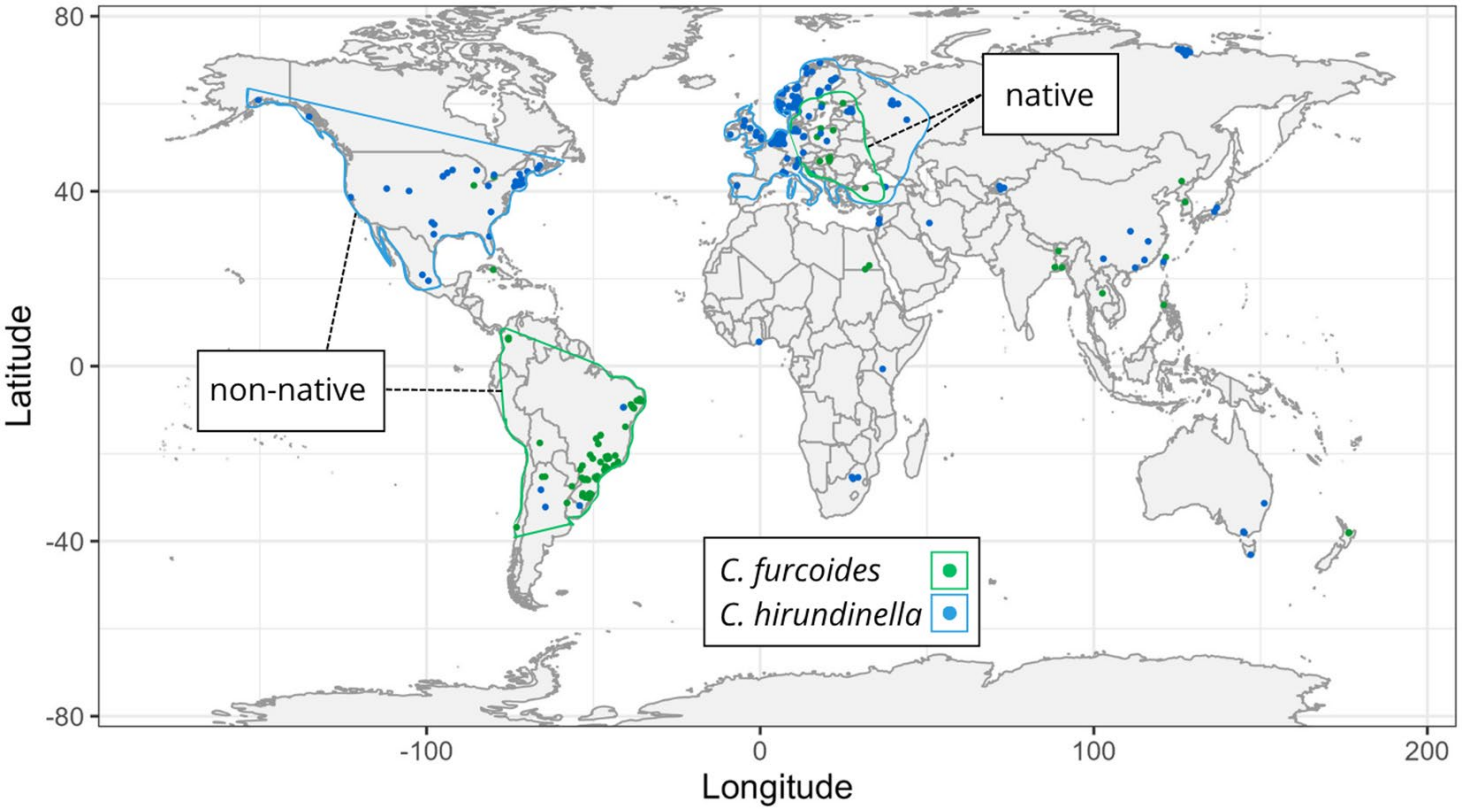
The environmental background for the niche analysis was defined using a minimum convex polygon (MCP) constructed from the occurrence records of each population group. This included the native range shared by both *C. furcoides* and *C. hirundinella*, as well as their non-native ranges.

The niche dynamics of the two *Ceratium* species were characterized following the method outlined by^42^. To assess niche conservatism and potential niche shifts, occurrence records for *C. hirundinella* (invasive mainly in North America) and *C. furcoides* (invasive mainly in South America) were divided into native and non-native regions. Background points were extracted using a minimum convex polygon (with an added buffer zone) defined for each region (native and non-native), from which the environmental niche space was estimated for each species (Fig. 1). To represent the environmental niche of the species, the climatic variables were reduced to a two-dimensional space using environmental principal component analysis (PCA-env). This PCA-env describes the environmental space, defined by the first two axes, that is available to both species across their ranges (Fig. 1). The species density in the environmental grid was then modelled based on observed occurrence density and the availability of environmental conditions in the background. A kernel density smoothing function was applied to the occurrences projected into environmental space, following^42^, to address potential biases from uneven sampling effort and spatial autocorrelation. This approach allows for a more accurate delineation of native and introduced niches by weighting environmental conditions according to occurrence density. To evaluate niche overlap, 10,000 pseudo-absence points were generated, and Schoener’s D overlap index was calculated 100 times to create a null distribution of overlap scores (α = 0.05)^43^.

To estimate niche overlap, which represents the area shared by the invaded and native niches, Schoener’s D overlap index was applied, ranging from 0 (no overlap) to 1 (complete overlap)^43^. A significantly low observed overlap suggests that the two occurrence sets are distinct, indicating that the species occupied different subsets of environmental space. The niche similarity test was used to compare the niche overlap of one range randomly distributed over its background while keeping the other unchanged (1→2), followed by a reciprocal comparison (1←2). For the niche similarity test, a p-value greater than 0.05 indicates that niches are less similar than expected by chance. Each randomization process was repeated 999 times, generating a null distribution of overlap values against which the observed score was compared. Niche changes were further decomposed into niche stability (the proportion of the invaded niche overlapping the native niche), niche unfilling (the proportion of the native niche not overlapping with the invaded niche), and niche expansion (the proportion of the invaded niche not overlapping the native niche)^7^. All analyses were performed using the ecospat R package in the R environment (R v4.2.2;^44^.

## Results

**Fig. 2:**
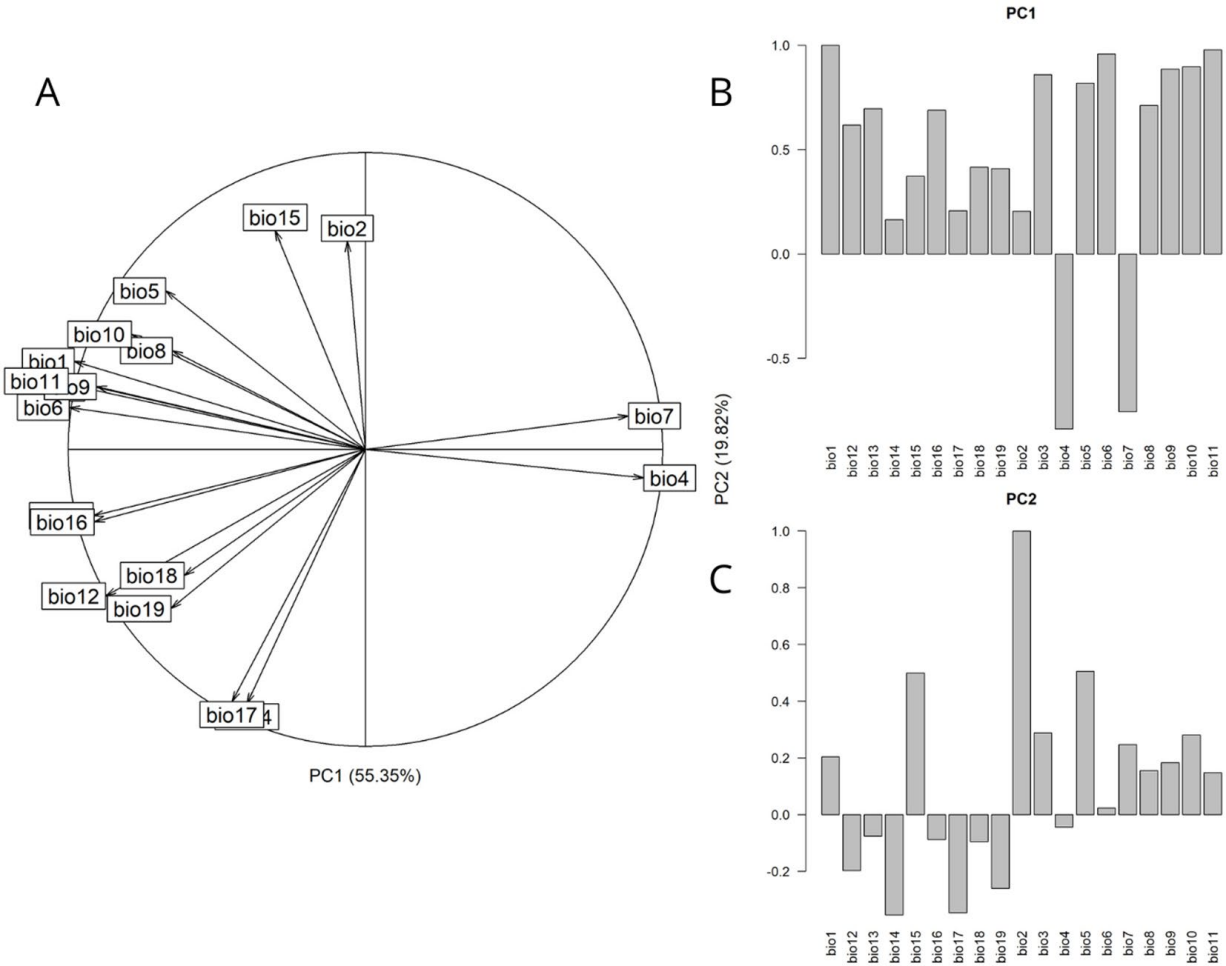
Principal Component Analysis (PCA-env) plot, illustrating the current distribution of *Ceratium* species in all its native and introduced ranges. (**A**) The first two PCA axes and the loadings (orientation) of each environmental variable on each axis. (**B**,**C**) Variable-specific contributions to the first and second PCA axes, respectively.

The first two PCA axes characterizing the environmental spaces explained 55.35% and 19.82% of the variation featured in the multivariate climatic dataset (Fig. 2A). The contributions of each variable to each of the two PCA axes are shown in (Fig. 2B,C). In PC1, the most influential variables were bio1 (annual mean temperature), bio4 (temperature seasonality), bio6 (minimum temperature of coldest month), bio10 (mean temperature of warmest quarter), and bio11 (mean temperature of coldest quarter). This axis primarily represents a temperature gradient associated with interannual thermal variability, ranging from warmer and thermally stable to cooler and seasonally variable environments. In contrast, PC2, was primarily associated with bio2 (mean diurnal temperature range), bio5 (maximum temperature of warmest month), bio14 (precipitation of driest month), bio15 (precipitation seasonality), and bio17 (precipitation of driest quarter). This axis reflects a precipitation and temperature variability gradient, particularly related to seasonal and inter-month climate extremes.

**Fig. 3:**
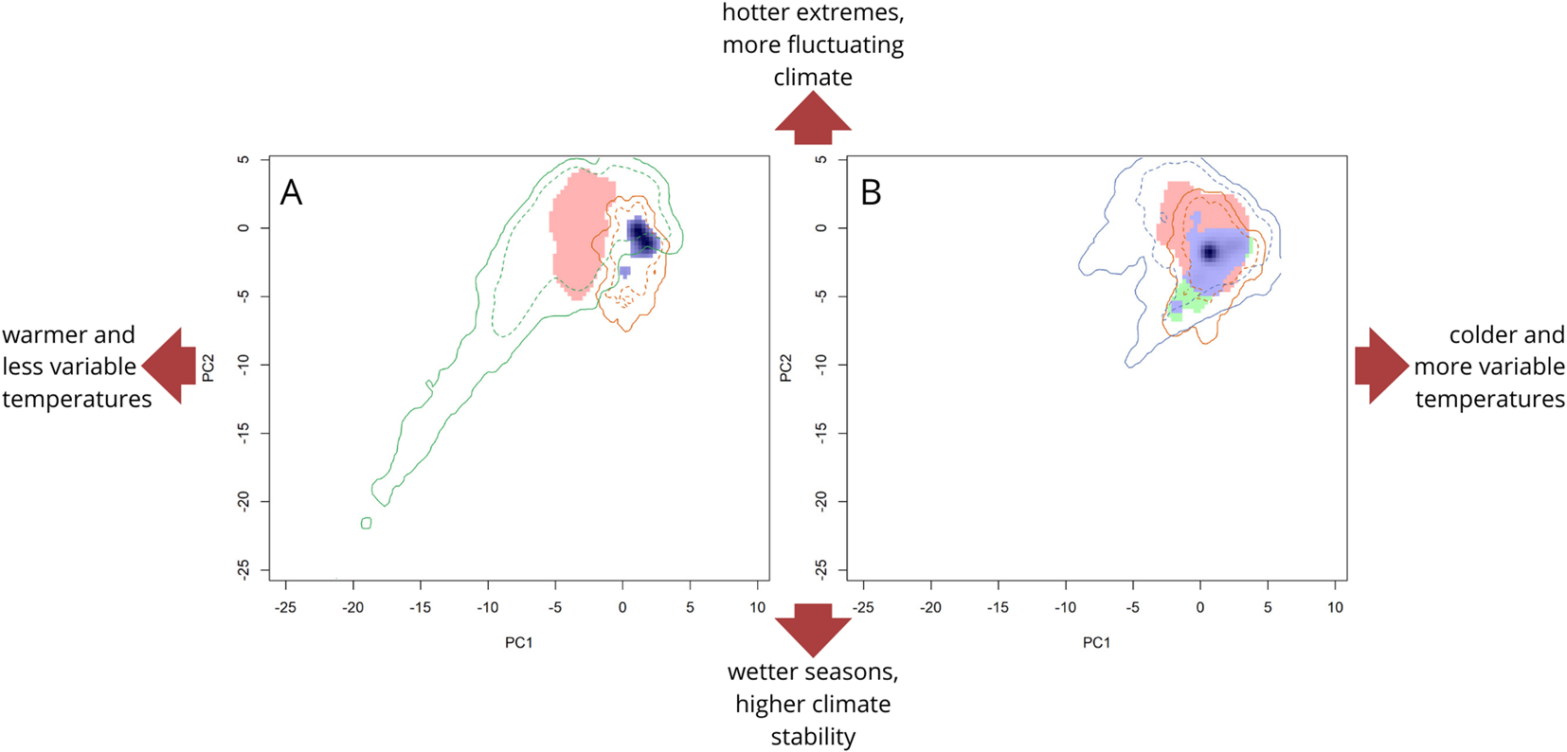
Niche overlap between native European (orange) and invasive south American (green) ranges of *Ceratium furcoides* (**A**) and native European (orange) and invasive north American (blue) of *Ceratium hirundinella* (**B**). Colored areas represent niche stability (blue), unfilling (green), and expansion (red). Solid and dashed lines delimit 100% and 75% of the available background environment considered in the analyses.

The ecological niche overlap (Schoener’s index) between the native and invaded ranges was 0.25 for *C. hirundinella* and 0 for *C. furcoides* (Table 1). For *C. furcoides* (Similarity Test; *p* > 0.05), the PCA-env indicated significant differences in its ecological niches between native and non-native ranges (Fig. 3A). Niches were more similar than expected by chance for *C. hirundinella* (Similarity Test; *p* < 0.01), indicating that the populations in the native and invaded areas have a highly similar environmental niche (Fig. 3B). According to this approach, only *C. hirundinella* presented high stability (96%), with similar environmental conditions occurring in both the native and invaded ranges. By contrast, *C. furcoides* showed both a maximum degree of niche expansion i.e., new environmental conditions occupied by non-native populations, and niche unfilling, i.e., the proportion of the native niche not occupied in the invaded areas following its invasion in South America. For *C. hirundinella*, in North America, there was a much lower degree of expansion (36%) and unfilling (4%).

**Fig. 4:**
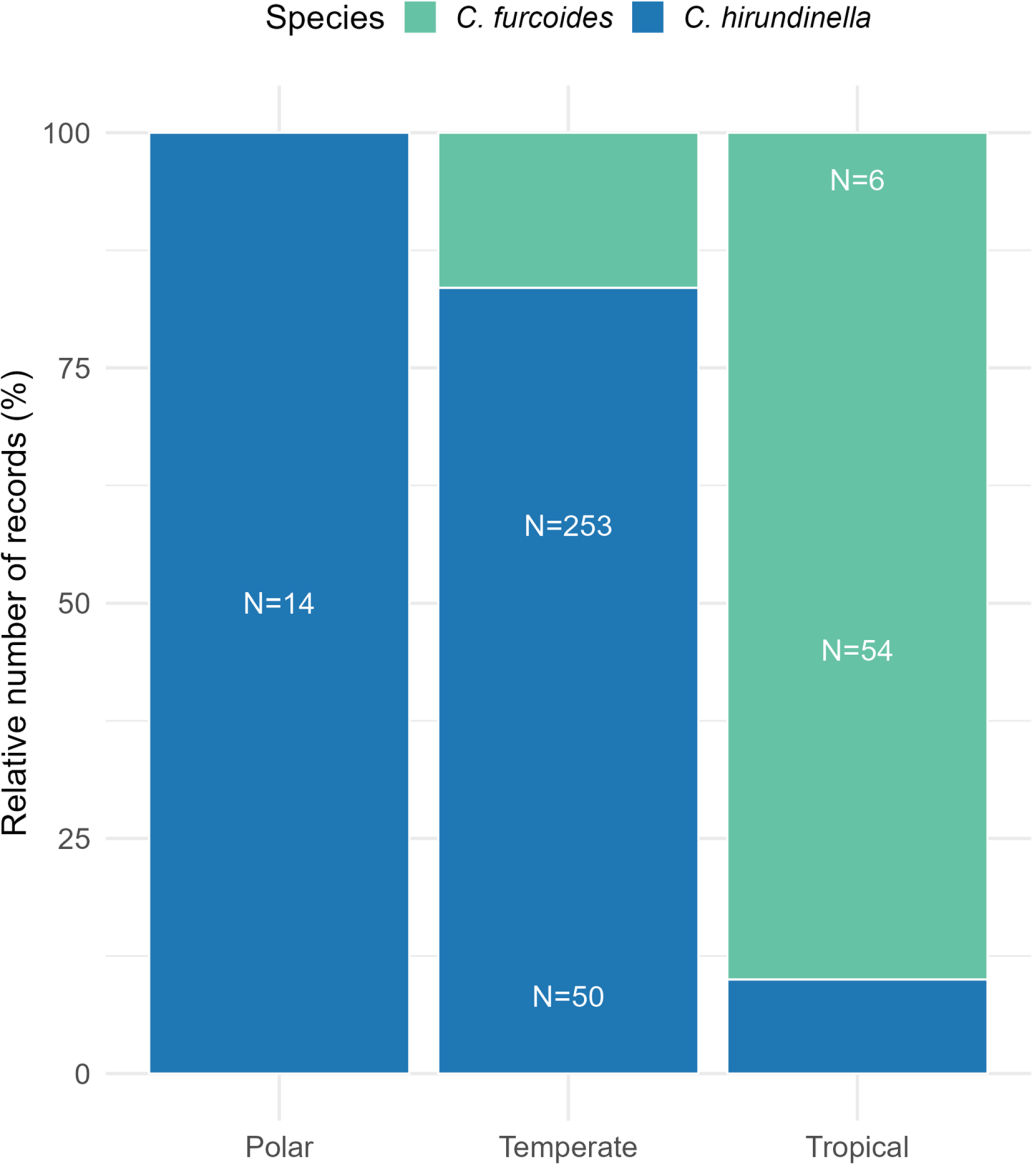
Relative number of GBIF records of studied congeners across different climatic zones (Polar, Temperate, Tropical). The height of each stacked segment indicates the relative contribution of each species. Total counts (N) of records for each species in each climatic zone are displayed within the bars.

The analysis shows distinct climatic preferences for the two species in their non-native ranges. Additionally, non-native populations of both species occupy broader environmental range than native ones (Fig. 3). Invasive populations of *C. furcoides* can thrive in environments characterized by greater precipitation seasonality, including drier climates. Conversely, although its native niche is a subset of the invasive niche, invasive populations of *C. hirundinella* are still strongly linked to regions with marked temperature variability across seasons. This pattern agrees with the relative distribution of records for *C. furcoides* and *C. hirundinella* across different climatic zones (Polar, Temperate, Tropical) based on GBIF data (Fig. 4), which show that *C. hirundinella* is predominantly found in temperate zones, while *C. furcoides* has a more even distribution across the temperate and tropical zones.

**Table 1:** Pairwise niche comparisons between the native and non-native ranges of *Ceratium* species. Note that the results from niche similarity and niche change metrics were tested reciprocally i.e., in both possible test directions.

	Overlap D	Similarity *p*-value		Unfilling %		Expansion %		Stability %	
		1➞2	2➞1	1➞2	2➞1	1➞2	2➞1	1➞2	2➞1
*C. furcoides*	0	1	1	1	1	1	1	0	0
*C. hirundinella*	0.25	**0.01**	**0.01**	0.04	0.36	0.36	0.04	0.64	0.96

## Discussion

The extent to which invasive species retain or shift their ecological niches during the invasion process remains a central question in biogeography and invasion ecology. While hundreds of species have been modelled to explore this dynamic^4,7,9^, microorganisms, particularly phytoplankton, remain markedly underrepresented in such analyses. This study addresses a key taxonomic gap in invasion ecology by building upon previous findings on *Ceratium furcoides*^25^, which reported minimal niche overlap between native and invasive populations based on Schoener’s D index. Expanding this previous finding, the present analysis investigates how invasive *Ceratium* congeners, species that often occupy similar fundamental niches, diverge in their realized niches after introduction.

*Ceratium furcoides*, in contrast to its congener *C. hirundinella*, showed a maximum niche shift following its invasion in the Neotropical region. In turn, the strong evidence of niche conservatism observed in *C. hirundinella* suggests that this species may be in a more stabilized phase of its invasion process, also evidenced by its lower niche unfilling—meaning it occupies only a subset of its realized niche from its native range within the introduced range. Conversely, the high niche unfilling in *C. furcoides* supports its higher occurrence probability in locations where it has not been recorded yet^26^, as well as its more recent introduction. While^26^ predicted future areas at risk of invasion by *C. furcoides*, such as regions in Africa, China, and Australia, further analyses are needed to assess whether these potential ranges will exhibit niche conservatism or further divergence. Notably, similar inconsistencies in climatic niche shifts have been previously reported for plant species across multiple introduced ranges^45,46^.

Darwin’s naturalization hypothesis^47^ posits that closely related species, due to overlapping ecological requirements, may encounter stronger biotic resistance in invaded communities, reducing their establishment success. In contrast, more distantly related species may face less competition and establish more readily. When applied to congeneric invaders, this framework raises the question of whether *C. furcoides* and *C. hirundinella*, despite their phylogenetic proximity and similar native habitats, have experienced divergent ecological pressures that encouraged markedly distinct realized niches in their invaded ranges. This observation supports earlier findings that phylogenetic relatedness does not necessarily predict ecological equivalence^2^.

Although the model species share certain morphological and ecological traits^48^, the environmental factors governing their establishment and bloom dynamics—particularly across space and time in invaded regions—remain poorly understood^23,49^. In their native range, both species overlap in terms of ecological requirements, typically occurring during the summer months (15–25 °C) in meso-eutrophic, stratified lakes with a slightly alkaline pH (ca. 8.0)^50^. However, this pattern seems to change in non-native ranges, where studies reported no or limited co-occurrence^19,51,52^. Given that eradicating such species is currently virtually impossible, interpreting the findings through the lens of ecological and evolutionary theories, particularly niche conservatism, has implications for managing invasions before they occur. This includes enhancing predictive models to map the full potential distribution of each congener separately, a task not yet accomplished for *C. hirundinella*, but also jointly. For instance, SDMs trained on both native and non-native range data^25^ identified additional high-risk areas for *C. furcoides* invasion along the shorelines of Uruguay, where blooms of this species were later associated with fish-kill events^12^. This area was not previously identified using presence solely from non-native ranges in South America^53^. Moreover, long-term monitoring programs are essential to predict not only blooms but also how environmental changes and different biological pressures affect co-occurrence patterns, plasticity, and invasion success at various phases of invasion by these species^26,54,55^.

This study is particularly relevant because the distribution of *Ceratium* species appears to be primarily shaped by environmental filters, such as temperature and water stability, rather than strong biotic pressures like predation or grazing, which are often lower for dinoflagellates compared to other phytoplankton groups^11,49,56,57^. In this context, while traits such as cyst production and sexual reproduction may enhance the invasive potential of *Ceratium* by facilitating persistence and dispersal across heterogeneous environments^58^, functional differences between *C. hirundinella* and *C. furcoides* may underlie their contrasting niche dynamics. For instance, phenotypic plasticity in this genus—largely morphological, as reflected in features like horn development and cell shape in response to environmental conditions^25,54,59^—likely contributes to their differential success in non-native environments. Additionally, mixotrophy—a strategy allowing organisms to combine photosynthesis with heterotrophic nutrition—has been reported in *C. hirundinella*^60^ and is well documented in several marine relatives^61–63^. In contrast, mixotrophy in *C. furcoides* remains unconfirmed and is considered controversial^63^. Although its potential occurrence cannot be entirely ruled out, the limited evidence for this trait in *C. furcoides* aligns with its expansion into regions with higher light availability.

The patterns observed in this study indicate that niche expansion was more pronounced than niche unfilling in both *C. hirundinella* and *C. furcoides*. This suggests that these species are not only tolerating novel environmental conditions but may be adapting or plastically adjusting to them. Such dynamics could result from rapid evolution, enhanced plasticity, or ecological release from competitors and natural enemies^64,65^. Nonetheless, little is known about their interactions with other planktonic taxa in non-native ranges, or the specific conditions under which colonization fails^21,48,66^. For instance, the high niche unfilling observed for *C. furcoides* might reflect competition with other dominant groups like cyanobacteria or native dinoflagellates. Physiologically explicit models and experimental studies are needed to validate these hypotheses and clarify the limits of their realized niches (see^57^.

Additional factors such as genetic drift or founder effects may also play a role by altering genetic structure during range expansion^67,68^. In the case of *C. furcoides*, an additional explanation for the observed divergence in niche occupation involves the possibility of rapid genetic differentiation. Identical or highly similar genetic sequences found in distinct non-native environments (see^69^ may not always represent the same ecological entity but could reflect recent radiation into cryptic species^70–72^. This would imply that phenotypic variation results from accumulated genetic differences caused by limited gene flow among geographically isolated populations. In dinoflagellates, this can even occur during blooms, (e.g., “sex for proliferation”;^73^, which could promote rapid local adaptation and increased phenotypic/genetic variability during invasion, contributing to niche expansion or divergence in invaded habitats. Such divergence may follow an initial phase of phenotypic plasticity—enabling rapid establishment across diverse environments—followed by adaptive differentiation driven by local selective pressures^74,75^. Over time, particularly in more stable or less climatically variable regions, the selective pressure to maintain plasticity may decline, allowing populations to specialize and stabilize within new ecological niches. The varying degrees of niche change observed in *Ceratium* are consistent with the idea that many phytoplankton taxa function as “collective species” (e.g^76^., comprising multiple ecotypes with divergent environmental tolerances and seasonal strategies^77–80^. This perspective supports the hypothesis that, rather than simply expanding their geographic range, *Ceratium* species may undergo local ecological differentiation when invading warmer, more hydrologically stable systems—resulting in distinct seasonal dynamics or dormancy adaptations. The contrasting niche dynamics observed between native and introduced ranges of *Ceratium hirundinella* and *C. furcoides* suggest an ecological ‘divide and conquer’ strategy during invasion. By adopting distinct colonization mechanisms and directions across non-native environments, these species may reduce direct competition and facilitate parallel establishment. Notably, each species has shown the greatest ecological impact within regions that align with their respective climatic optima — with *C. hirundinella* well-established and impactful in temperate regions^10,81^ and *C. furcoides* in tropical and subtropical^12,26^. Although this study did not directly evaluate this process, such spatial and ecological divergence may promote long-term coexistence, as local selective pressures reinforce niche differentiation and reduce competitive exclusion over time.

Finally, this study suggests that dinoflagellate invasions may involve more complex ecological and evolutionary mechanisms than previously recognized. In this context, risk assessments must adopt adaptive, region-specific, and population-level approaches to account for ecological variability and differential susceptibility^82^. Overall, the invasion trajectories of *Ceratium* species are likely shaped by a combination of eco-evolutionary dynamics, invasion history, and habitat filtering^5,6^, and as such, it is essential that researchers and managers routinely quantify niche changes when modeling the potential distributions of rapidly evolving invasive species, in order to improve risk assessments and deepen our mechanistic understanding of invasion processes and impacts.

## Supplementary Information

Below is the link to the electronic supplementary material.

Supplementary Material 1

## Data Availability

Data used in this study are available as supplementary material.
